# Caldera unrest driven by CO_2_-induced drying of the deep hydrothermal system

**DOI:** 10.1038/s41598-018-26610-2

**Published:** 2018-05-29

**Authors:** R. Moretti, C. Troise, F. Sarno, G. De Natale

**Affiliations:** 1 Dipartimento di Ingegneria, Scuola Politecnica e delle Scienze di Base, Università degli Studi della Campania “Luigi Vanvitelli”, Via Roma 29, 81031 Aversa, CE Italy; 2 Istituto Nazionale di Geofisica e Vulcanologia, sezione di Napoli Osservatorio Vesuviano, Via Diocleziano 328, 80124 Napoli, Italy; 3 Present Address: Institut de Physique du Globe de Paris – équipe Systèmes Volcaniques, 1 rue Jussieu, 75238 Paris, cedex 05 France

## Abstract

Interpreting volcanic unrest is a highly challenging and non-unique problem at calderas, since large hydrothermal systems may either hide or amplify the dynamics of buried magma(s). Here we use the exceptional ground displacement and geochemical datasets from the actively degassing Campi Flegrei caldera (Southern Italy) to show that ambiguities disappear when the thermal evolution of the deep hydrothermal system is accurately tracked. By using temperatures from the CO_2_-CH_4_ exchange of ^13^C and thermodynamic analysis of gas ascending in the crust, we demonstrate that after the last 1982–84 crisis the deep hydrothermal system evolved through supercritical conditions under the continuous isenthalpic inflow of hot CO_2_-rich gases released from the deep (~8 km) magma reservoir of regional size. This resulted in the drying of the base of the hot hydrothermal system, no more buffered along the liquid-vapour equilibrium, and excludes any shallow arrival of new magma, whose abundant steam degassing due to decompression would have restored liquid-vapour equilibrium. The consequent CO_2_-infiltration and progressive heating of the surrounding deforming rock volume cause the build-up of pore pressure in aquifers, and generate the striking temporal symmetry that characterizes the ongoing uplift and the post-1984 subsidence, both originated by the same but reversed deformation mechanism.

## Introduction

Caldera unrest is a complex phenomenon consisting in a departure from the background or baseline behaviour of geophysical and geochemical indicators, such as changes in ground level, seismicity, gravity and degassing (total discharge, CO_2_ emissions and changes in gas chemistry) due to magma and/or hydrothermal system dynamics^1–3^. However, large uncertainties in the knowledge of the links between hydrothermal and magmatic processes affect the forecasting of the unrest evolution. Such uncertainties in determining the source and likely outcome of the unrest are even larger for calderas than for other volcanoes, essentially because of the stronger influence of hydrothermal systems at calderas^2–4^. Ground uplift, a typical marker of caldera unrest, may reflect magma movement and pressurization as well as pore pressure increase due to the changes in fluid phase and hydraulic flow patterns^5–11^. Variations in gas discharge are commonly perceived to represent a delayed response to magmatic changes, being mainly controlled by the hydrothermal system and rock permeability^8–12^. Furthermore, interpretations of ground deformation and geochemical data are too model-dependent and very often contrasting, such as in the case of determining the nature, size and depth of the overpressure source causing deformation^13,14^.

**Figure 1:**
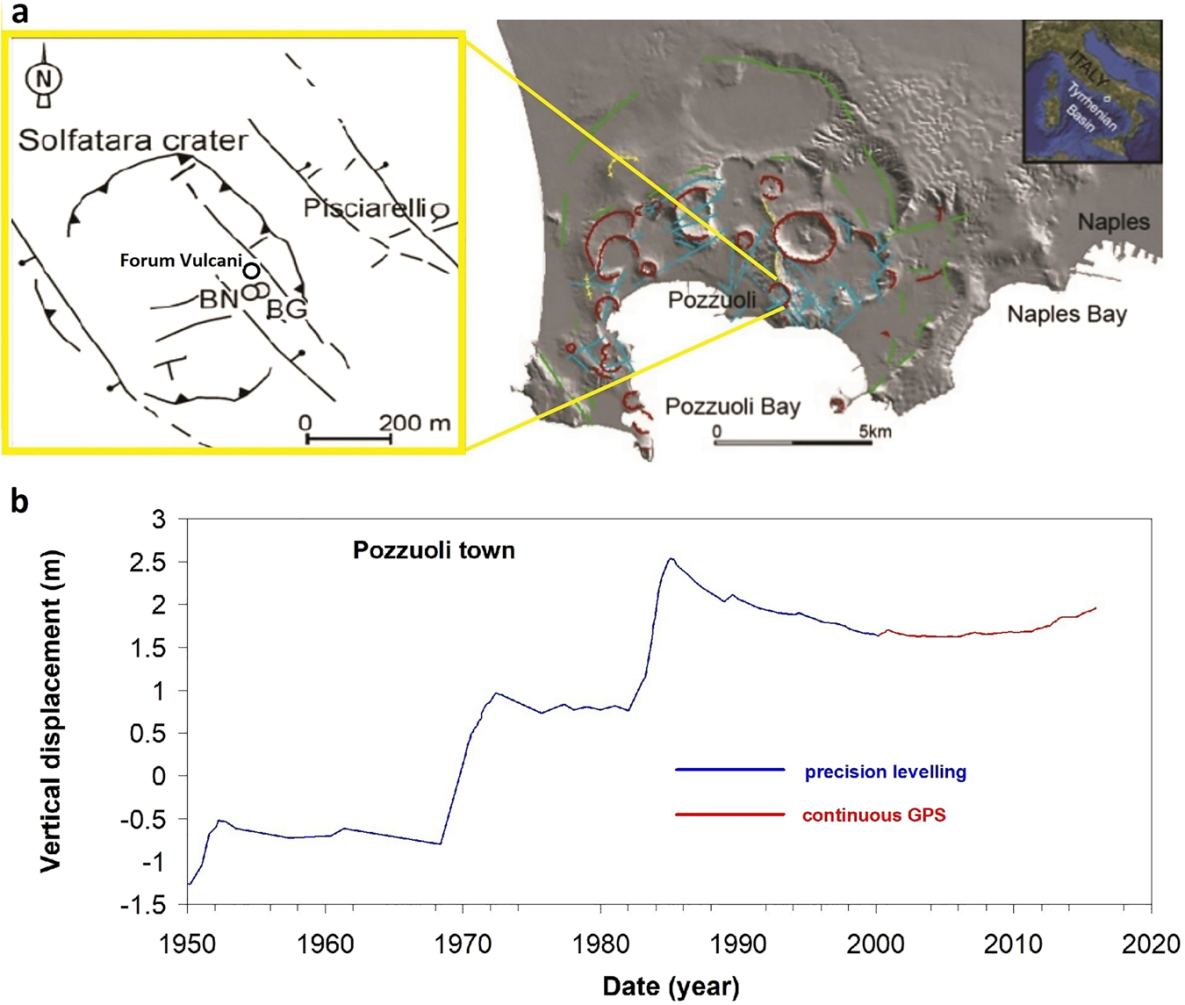
CFc setting and long-term ground displacement. (**a**) Location and structural sketch of Campi Flegrei caldera. Also reported are locations of (2) present Solfatara main fumaroles, Bocca Grande (BG), Bocca Nuova (BN), plus Forum Vulcani, which collapsed during the 1982–84 unrest, and (2) the Pisciarelli fumarole (**b**). Chronogram of the maximum vertical ground displacement^15,19^. Sharp uplifts characterized unrest episodes occurred in 1950–52, 1969–72 and 1982–84. A 20 year-long subsidence took place at the end of the 1982–84 crisis. A new unrest phase started around year 2005, characterized by a lower uplift rate than during the previous uplift episodes.

Ambiguities and difficulties in understanding the role of magmatic and hydrothermal triggering processes are well illustrated by the non eruptive unrest occurring at Campi Flegrei caldera (CFc) for decades (Fig. 1). CFc is a large (~12 km in diameter), restless caldera surrounding the Neapolitan area (Southern Italy), exposed to the highest volcanic risk in the world given the intense urbanisation^15–17^. Its evolution in historical times is marked by the Monte Nuovo eruption^18^, the last one, occurred in 1538 after a repose period of circa 2500 years, and by the continuous alternation of ground uplift and subsidence phases^15,16,19,20^. A vigorous hydrothermal system, fed by magmatic gases, drives the diffuse degassing of huge amounts of CO_2_, fumarole activity and mud pools^14,15,21–24^.

Here, we discriminate uplift episodes driven by magma intrusion at shallow depth from those driven by deep fluid influx in the aquifers, taking advantage of the exceptional, more than 35-years long, CFc geochemical record of CO_2_-rich gases discharged from fumaroles located within the Solfatara crater (Forum Vulcani, Bocca Grande, and Bocca Nuova vents) and the Pisciarelli area^15,21–24^ (Fig. 1a). Our approach is based on the reconstruction of the thermal evolution and phase changes of the deep hydrothermal system, independently recorded by carbon isotopic fractionation, and on thermodynamic analysis of CO_2_-rich ascending hydrothermal fluids. Combined with a model-independent description of ground-deformation patterns, and contrary to other recent model outcomes^25^, this analysis unravels the non-magmatic nature of the ongoing CFc unrest, due to supercritical excursion of the deep hydrothermal system. Our model-independent approach can also be applied widely to determine the source of unrest and its temporal evolution at other calderas.

## Results

### Non-eruptive unrest at the Campi Flegrei caldera

The presently ongoing CFc uplift episode occurs after ~20 years of subsidence that followed the 1984 peak ground uplift, when a maximum of ~4.5 m cumulative vertical displacement occurred over three major unrest episodes since 1950 (Fig. 1b). For this reason it is raising serious concern, such that since 2012 Civil Protection issues a volcanic alert of level 2 (‘Attention’) on a scale of four. Although the maximum uplift attained in 1984 was recognized to led the system very close to the purely fragile behaviour, the subsequent subsidence (Fig. 1b) clearly represents an anomalous behaviour with respect to other volcanoes, such as Rabaul caldera^26^, drawing back the system away from the critical rupture point^26^. It is then of paramount importance to establish the actual nature of the ongoing uplift, as compared to the 1982–1984 episod, in order to assess its hazard implications.

**Figure 2:**
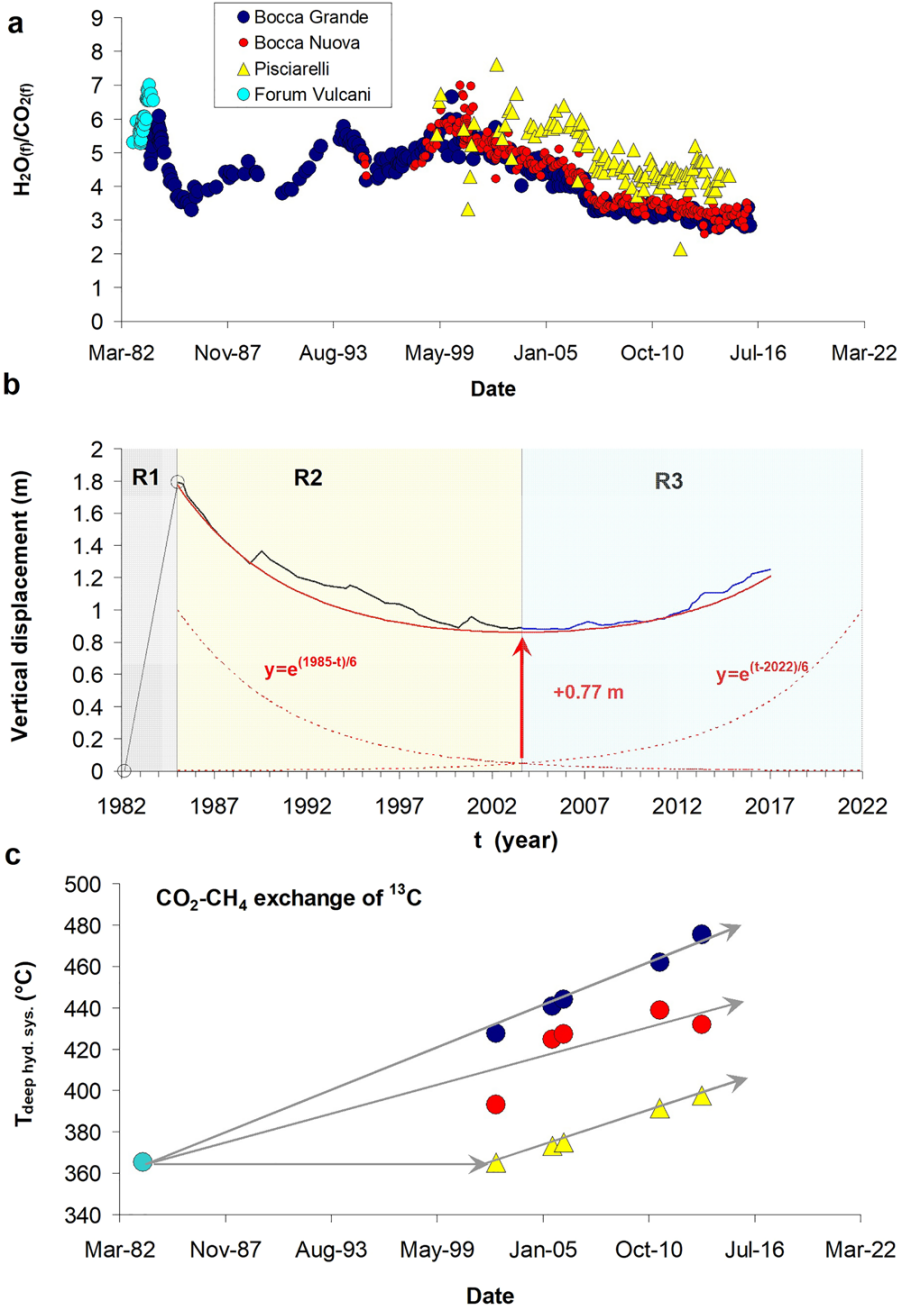
Major geochemical and ground deformation features since 1982. (**a**) Chronogram of H_2_O/CO_2_ (molar ratio) at Solfatara (Forum Vulcani, Bocca Grande and Bocca Nuova vents) and Pisciarelli fumaroles (Supplementary Data and refs^15,22–25^). (**b**) Baseline displacement (red-solid line) involving the post-1984 subsidence and the ongoing unrest (the zero is set at the beginning of the 1982-84 uplift). Error bars on measured displacements are ±4 mm for leveling (until year 2000; ref.^19^) and ±3 mm for continuous GPS (after year 2000; from INGV-OV surveillance bulletins: http://www.ov.ingv.it/ov/en/campi-flegrei/275.html). Three deformation regions are distinguished (R1, R2 and R3). (**c**) Chronogram of CO_2_-CH_4_ isotopic temperatures at the hydrothermal bottom (see Methods and Table 1). Heating trends (grey arrows) show the drying of the hydrothermal system.

During the 1982–84 episode, H_2_O/CO_2_ increased rapidly up to ~7 in 1983 and then abruptly decreased to ~4 at the apex of ground deformation in 1984; it then attained a new maximum of ~7 in year 2000, to then decrease smoothly down to ~3 in the present unrest (Fig. 2a). This contrasting geochemical behavior requires triggering mechanisms that are different for the 1982–84 and the ongoing unrest episodes, and highlights that if unrest were magmatic in 1982–84, it must be hydrothermal now^15,27^. It was then proposed that decreasing H_2_O/CO_2_ ratio in post-1984 fumarole gases reflects the progressive arrival of hot gas, with H_2_O/CO_2_ between 1 and 1.5, released from the deep (8 km) magma and percolating through the shallow and permeable 1982–84 magma, which around 2000 exhausted its gas and solidified^15,23^. In an alternative model, gas signatures would be compatible with a magma progressively decompressing since 1984 and releasing gases with increasing H_2_O/CO_2_ ratio^24,25,27^. The subsequent condensation of large amounts of steam within the hydrothermal system would be invoked, in this hypothesis, to explain why a concomitant decrease of the H_2_O/CO_2_ ratio is observed in Solfatara and Pisciarelli fumarole fluids (Fig. 2a). Such a process would progressively heat up the hydrothermal reservoir rocks and increase pore pressure since 1984, thus triggering an accelerating deformation that could culminate in rock failure and eruption on attainment of a critical decompression threshold of the degassing magma^26^. Despite the different geochemical signatures and interpretations, we observe here for the first time that a striking temporal symmetry characterizes the evolution of ground displacement after the 1984 apex: two mirror exponential functions create a symmetric pattern of ground displacement whose minimum (0.86 m) is located in mid-2003 and is mainly due to a permanent deformation (0.77 m) residual from the 1982–84 unrest (Fig. 2b). This permanent deformation is very likely due to magma emplaced in 1982–84, still molten or eventually solidified^23,28^, but it may also represent a residual deformation of aquifers under drained conditions^29^. Subsidence recovered ~1 m of the maximum 1982–84 uplift (1.8 m) and led the system towards the 2003 bottom value. In the period 2000–2005 subsidence stopped, and a new uplift period started. Both subsidence and ongoing uplift have a symmetric baseline that can be described by means of a simple exponential law, having the same argument, changed of sign, and opportunely shifted in time to account for relative contributions of deflation and inflation in reproducing the main trend of vertical displacement (Fig. 2b). The fact that the involved rock volume experiences deflation and then inflation following mirror mechanisms is a powerful constraint to detect the nature of unrest, and is a further observation excluding any arrival of new magma after 1984. It also suggests that the deforming rock volume preserves the same mechanical properties both in the subsidence and in the ongoing uplift phases (Fig. 2b).

### Thermal features and evolution of the hydrothermal system

The Solfatara-Pisciarelli deep hydrothermal system was earlier interpreted as a liquid + vapor system located close to the critical point of water, at P_fluid_ ≈ 25 MPa, or PH_2_O ≈ 20 MPa, and ~360 °C (ref.^22^). It is fed by hot magmatic gases (~900 °C) that vaporize and mix with the pure hydrothermal (meteoric) component^22^. This interaction produces the final CH_4_-bearing fluid plume which formation temperature is recorded by the CO_2_ and CH_4_ isotopic exchange^21,23,30,31^ (Fig. 2c and Table 1; see Methods). However, by considering such isotopic temperatures we observe a progressive heating of the deepest part of the hydrothermal system, above the critical point of water. The only data available before 2002 (365 °C, measured in Forum Vulcani fluids in 1983) allow tying the entire thermal evolution to an initial condition corresponding to the 1982–84 unrest^21^, when CH_4_ clearly formed in a boiling aquifer very close to the critical point of water. Because fluids discharged at Solfatara and Pisciarelli point to the same deep physico-chemically homogenous hydrothermal system^21,22^, this initial condition is common to all fumarole systems in these areas.

**Table 1:** Data used to compute the isotopic temperature from ^13^C exchange between CO_2_ and CH_4_ (see Methods).

Date	Fumarole	δ^13^CCO_2_	δ^13^CCH_4_	Δ^13^CCO_2_-CH_4_ (1000lnα)	T (°C)	Ref.
6/16/1983	Forum Vulcani	—	—	21.05	365.2	^21^
7/15/2002	BG	−1.31	−19.2	17.89	427.6	^22^
8/2/2005	BG	−1.47	−18.8	17.33	440.8	^22^
3/8/2006	BG	−1.3	−18.5	17.2	444	^22^
5/18/2011	BG	−1.1	−17.6	16.5	462	^30^
9/2/2013	BG	−1.2	−17.2	16	475.5	^31^
7/15/2002	BN	−1.08	−20.6	19.52	393.2	^22^
8/2/2005	BN	−1.58	−19.6	18.02	424.7	^22^
3/8/2006	BN	−1.5	−19.4	17.9	427.3	^22^
5/18/2011	BN	−1.1	−18.5	17.4	439	^30^
9/2/2013	BN	−0.8	−18.5	17.7	432	^31^
7/15/2002	Pisciarelli	−1.35	−22.4	21.05	365	^22^
8/2/2005	Pisciarelli	−1.52	−22.1	20.58	373.4	^22^
3/8/2006	Pisciarelli	−1.62	−22.1	20.48	375.1	^22^
5/18/2011	Pisciarelli	−1.2	−20.8	19.6	391.5	^30^
9/2/2013	Pisciarelli	−1.2	−20.5	19.3	397.5	^31^

The subsequent evolution up to 476 °C (Table 1) is due to the progressive drying of the boiling low-saline liquid water at the bottom of the hydrothermal system after 1984 and since around year 2003 at Pisciarelli. The observed deep heating is a strong argument against the presence of a shallow magma injecting increasing amounts of steam that subsequently condense (see Supplementary Information). In fact, steam condensation should keep the deep portion of the hydrothermal system thermally buffered along the liquid-vapor coexistence originally close to the critical point of water^22^. On the contrary, the dried hydrothermal low-saline liquid has been replaced by a high-T vapor or even supercritical gas (see Supplementary Information). This easily explains the decreasing H_2_O/CO_2_ ratio in fumarole gases and the relative increase of the gas magmatic fraction (>0.4) (refs^22,23^), as well as the high gas fluxes at surface^14,24^.

**Figure 3:**
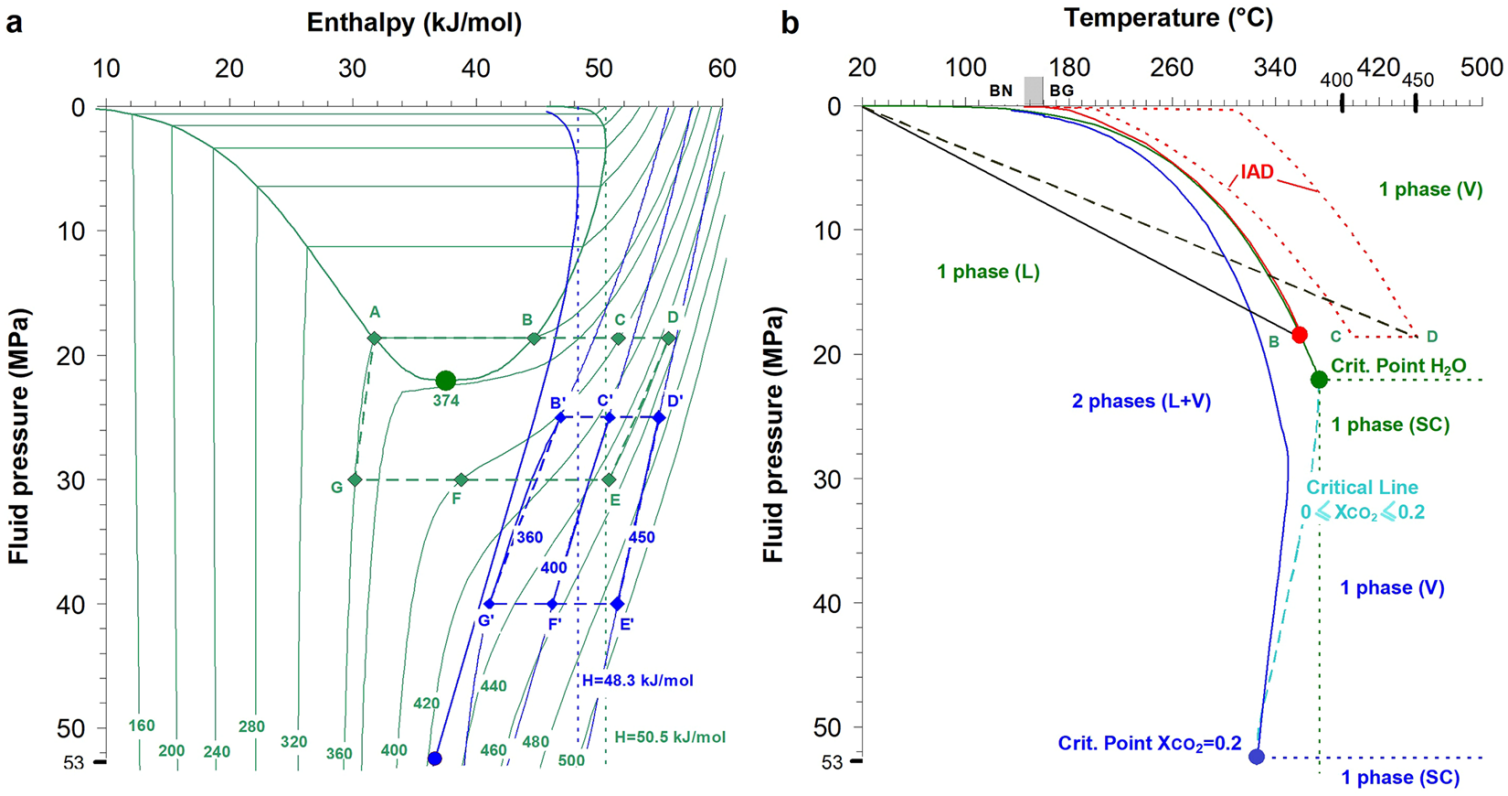
P-H-T properties of the hydrothermal system. (**a**) P_fluid_-H diagrams for pure water (thin green lines: isotherms; thick green line: vapour-liquid equilibrium) and for a H_2_O-CO_2_ 80:20 (mol) mixture (thin blues lines: isotherms; thick blue line: vapor-liquid equilibrium-dew line). Green and blue labels refer to pure water and H_2_O-CO_2_ mixture, respectively. Numbers labeling isotherms refer to °C. See Methods for source of data. (**b**) Vapor-liquid equilibria and phase fields for pure water (green line and green labels) and for the 80:20 H_2_O-CO_2_ mixture (blue line and blue labels), together with the critical line for intermediate fluid compositions (turquoise line). L: Liquid; L + V: coexisting liquid and vapor; SC: supercritical gas. Also shown (with respect to pure water only, for the sake of graphical clarity) are: 1) the P-T path of the 1982-84 fluid plume (solid red line), 2) the locus of condensation-free ascent fluid paths between 2001 and 2013 (enveloped by the dotted closed red line), and 3) the P-T paths outside the hot fumarolic area (solid and dotted black straight lines). See Methods for sources of data.

Pressure-temperature-enthalpy (P_fluid_-T-H) paths (Fig. 3; see Methods) encountered in the subsoil of the fumarole system and of the area of maximum deformation from 1984 to present, shows that the hydrothermal system bottom is presently too hot to supply a gas condensing on its rise toward surface along the fumarole channel, which at first approximation is isenthalpic (Fig. 3a,b), corresponding to an irreversible adiabatic decompression (IAD) (see Methods). In Fig. 3a we plot P-H properties of pure water and a (molar) 80:20 H_2_O-CO_2_ real mixture (critical point at 52.5 MPa and 326 °C; H_vapor_ = 36.7 kJ/mol; ref.^32^), which is a good average of the last 10-year discharges (Fig. 2a). If we take as reference pure water (in green in Fig. 3a), upon IAD, condensation cannot occur for H > 50.5 kJ/mol, thus the present-day deep hydrothermal gas (~450 °C) would condense only if its initial (water) pressure rises from ~20 MPa (ref.^22^) to ~30 MPa. If we consider, the 80:20 H_2_O-CO_2_ real mixture (in blue in Fig. 3a), upon IAD, condensation cannot occur for H > 48.3 kJ/mol, such that the present-day deep hydrothermal gas at 450 °C would not condense during isenthalpic expansion (unless starting IAD from pressure unreasonably high for the CFc system, i.e. >50 MPa; Fig. 3a).

With reference to pure water, the ABCDEFG quadrangle (Fig. 3a) then surrounds the domain of bottom hydrothermal conditions for enthalpies corresponding to temperatures increasing from 360 °C to 450 °C, as required by the CO_2_-CH_4_^13^C exchange (Fig. 2c and Table 1) and pressures of interest (PH_2_O from ~20 to 30 MPa). Assuming at first approximation the validity of the Raoult’s law, the pressure excursion at the base of the hydrothermal system would become ~25 MPa to ~40 MPa for the 80:20 H_2_O-CO_2_ fluid, thus defining, for the same T-interval, the B’C’D’E’F’G’ quadrangle (Fig. 3a). It is worth noting that in both cases, the pressure increase (10 MPa for pure H_2_O and 15 Mpa H_2_O-CO_2_ mixture) does not exceed the threshold imposed by CFc rock tensile strength^13^.

The pure-H_2_O ABCDEFG quadrangle (Fig. 3a) embraces liquid, vapour and supercritical phases, and shows that the deep hydrothermal system has evolved as a supercritical fluid around the critical point of pure water. With reference to the 80:20 H_2_O-CO_2_ mixture, we see that the deep hydrothermal gas (B’C’D’E’F’G’ quadrangle; Fig. 3a) would be directly placed in the superheated vapour region. Therefore, accounting for the H_2_O-CO_2_ mixture, rather than pure water, expands the range of conditions for which liquid condensation cannot be encountered. This allows us stating that gas emissions associated with the ongoing unrest, and more generally with the post-1984 subsidence (Fig. 2a), result from a single-gas expansion, from the base of the hydrothermal system upward. On the other hand, the 1982-84 gas has evolved by condensing either water, or saturated H_2_O-CO_2_ vapour, as shown by independent P-T estimates of the last equilibrium recorded by 1982–84 fumarolic gases (Supplementary Figure 1), which are compatible with either the point of maximum enthalpy of the saturated steam (236 °C, 3.1 MPa) or the saturated H_2_O-CO_2_ vapor (~250 °C and ~5 MPa).

These features can be appreciated in Fig. 3b, which reports the P-T curves for pure water and for the 80:20 H_2_O-CO_2_ mixture, the critical line for intermediate H_2_O-CO_2_ mixtures, as well as the P-T region (with reference to steam) within which Solfatara fluids evolve from PH_2_O ≈ 20 MPa and temperatures between 400 °C to 450 °C (CD line in Fig. 3b) up to surface fumaroles. Differently from the 1982–84 fluid plume, which condensed up to the point of maximum enthalpy of the saturated steam (solid red line), fumarole fluids discharged between 2001 and 2013 (enclosed within the dotted red line, Fig. 3b) do not condense and can reach, by IAD (isenthalpically), pressures up to PH_2_O ≈ 0.3 MPa and temperatures between 210 °C and 300 °C. They then reach fumarole outlets at ~160 °C (BG) and ~145 °C (BN, as well as Forum Volcani, collapsed in 1984) (gray box along the X-axis in Fig. 3b), vent temperatures being fixed by the gas flow energy conservation throughout the terminal section of fumarolic conduits^33^.

In agreement with geothermal gradients measured at CFc drilling sites^34^, Fig. 3b also shows the evolution of the P-T paths originating from initial bottom temperatures of 360 °C (solid black line representing the 1982–84 path) and 450 °C (dashed black line, representing attainment of steady state conditions). These are external to the fumarole field but internal to the area of maximum deformation and intersect the sequence of aquifers that saturate the rock medium accommodating the whole deformation on top of the deep hydrothermal system. The Solfatara and Pisciarelli fumarole fields are located along structural discontinuities (fault planes) that probe the physico-chemical evolution of the hydrothermal reservoir, spilling directly its deepest portion. Solfatara and Pisciarelli fumaroles are located along the same axis at 1.8 and 2.3 km, respectively, north-east from Rione Terra (Pozzuoli downtown), where maximum vertical displacement is observed. Fumaroles are a window on the deep hydrothermal system, but horizontally offset from the area of the maximum deformation that is modulated by the P_fluid_-T evolution of aquifers on top of the drying hydrothermal system. Considering a uniform bottom heating up from 360 °C to 450 °C, the P-T path crossing aquifers should progressively vary until reaching a new steady state (dashed black line, Fig. 3b), still enclosed within the liquid region. Despite the temperature of the hydrothermal bottom zone increased of ~90 °C in around 30 years (Figs 2c and 3b), thermal diffusivity analysis (*κ* = *k/ρCp*; with *k* = 2.8 W/m·K and *Cp* = 1000 J/kg·K, *ρ* = 2000 kg/m^3^) (refs^35,36^) shows that the new steady state geothermal gradient through aquifers has not been reached yet, and that heat conduction alone may have propagated the thermal anomaly to a distance $\lambda \approx 2\sqrt{\kappa \tau }$ of 73 m above the ~2.5 km-deep base of the hydrothermal system (*τ* = 30 years). On the other hand, the efficient advective transport of heat in fumaroles produced, since 2007, a T-increase of 15 °C in the very shallow (<0.5 km-deep) single gas equilibrium zone^15^ (Supplementary Figure 1). Therefore, outside the hot fumarole area, in the water-saturated porous medium hosting the main aquifers of the deformed area, where heat transport is neither purely advective nor conductive, we should expect at present an average thermal increase of few degrees (<5 °C). This small increase is determined by the permeability-driven slow infiltration of hot CO_2_ and its dissolution in groundwaters and is consistent with the comparison of results at different time-steps^37,38^ from numerical simulations of the CFc thermal field, which is dominated by the advective flux of CO_2_-rich hot fluids.

It is worth noting that based on 2D simulations of the only conductive and convective heat transfer resulting from the sudden sill emplacement at 4 km depth, we can, in fact, rule out any appreciable effect related to the long-lasting conductive thermal release of the 1982–84 sill intrusion on the surrounding rocks and the base of the hydrothermal system (see Methods and Fig. S2). In addition, simulations returns rapid cooling below solidus, in the order of ~10 years, confirming previous findings^15,23,28^ and further marking the impossibility that a shallow magma emplaced in 1982–84 may supply fluids until nowadays and even further (see Supplementary information). Therefore, the thin (<0.8 m; Fig. 2b) 1982–84 intrusion cannot have determined the progressive T-increase over time, as inferred from geochemical data. The very rapid cooling and the lack of a a long-lasting thermal effect associated with the thin-sill intrusion requires that a different heating source, associated with the rise of CO_2_-rich deep fluids, has 1) dried over time the base of the hydrothermal system and 2) determined, in the shallow aquifers, thermoelastic effects that are reponsible of the ongoing ground displacement (Fig. 2b).

## Discussions and Conclusions

The 1982–84 unrest was determined by the shallow intrusion of a sill-like magmatic body, that suddenly injected hot steam-rich fluids into the hydrothermal reservoir^23^. Steam condensation determined the shallow hydrothermal heating (up to 240 °C; Supplementary Figure 1 and ref.^15^) and contributed to the rapid 1982–84 uplift via undrained expansion^29,39^ (R1 in Fig. 2b). The intrusion thickness shoud approximate quite well the permanent deformation of ~0.8 m (Fig. 2b), but we cannot exclude that a residual thermoelastic deformation of aquifers^29^ contributes to the latter.

The 20-year long subsidence of ~1 m after the 1984 peak (R2 in Fig. 2b) can only be explained via relaxation of the hydrothermal system and aquifers, which soon restored drained conditions^29,39^ by rapidly releasing the excess hot fluids and progressively dissipating pore pressures. Solfatara fumaroles record this process by the decrease of shallow hydrothermal temperature and the rapid drop of pressures, already started in 1984 (Supplementary Figure 1).

Around year 2000, upon solidification and fracturing of the shallow sill emplaced in 1982, continuous percolation of the deep (8 km) magmatic gases replaced the previously pulsating behaviour, as shown by the H_2_O/CO_2_ ratio (Fig. 2a). This process was accompanied by the increase in seismicity observed since year 2000, also showing 3-4 km deep hypocenters (see Methods and ref.^40^) that can be related to the ongoing fracturing within the solidified magma sill. The observed drying of the deep hydrothermal component was then caused by the heat supplied by the CO_2_-rich gas, on its ascent from the deep degassing magma (200 MPa and 1000 °C) (refs^23,41–43^) to the bottom of the hydrothermal system at 25 MPa (see Methods and Supplementary Figure 2). Around year 2003, the deep hydrothermal drying became effective at Pisciarelli too, and likely involved most of the aquifers of the deformed area. Then a new uplift phase became evident, reversing the previous scenario (R3 in Fig. 2b). The ongoing uplift should be then ultimately caused by infiltration of deep CO_2_-rich hot gases into the hydrothermal system and aquifers with subsequent temperature increase and pressure relaxation in response to the upward propagation of the deep thermal anomaly (Fig. 2c). Consistent with this, fumarole gases display an increasing equilibrium temperature (since 2007) and a constant equilibrium pressure, close to the atmospheric one since 2005 (Supplementary Figure 1 and ref.^15^), attained along nearly-isenthalpic ascent paths, sketched by the violet arrow in Fig. 3a and enveloped by the red dotted area in Fig. 3b.

In a porous medium expansion of the water + gas filled rock volume occurs in response to changes of temperatures within the reservoir and involves a volume in the range 15–35 km^3^ (considering the present uplift of 40 cm; Fig. 2b) (see Methods). We do not model here the time dependence of ground deformation (Fig. 2b), which for hydrothermal unrest is well-established even for fixed, constant source parameters^8–11^. However, our analysis points out that the ongoing unrest is restoring, partially or totally, the same conditions that in 1984 led the system very close to the critical fragile behavior, which is the critical point when an eruption becomes very likely^25,41,42^. Therefore, despite the ongoing ground deformation not being linked so far to magma migration to shallow depths, the unrest could switch to a phase of magma ascent^43–45^: our findings in fact suggest that a chemical window is opening over the deep magmatic system, and that direct connections with it may be activated, depending on the accumulated stress and the attainment of the fragile threshold for rock fracture^26^. We speculate here that the onset of such connections may explain why the CFc magma reservoir could be activated at any time^44^, leading to explosive behaviour on a short timescale of hours or days^46,47^.

Given the present poor knowledge of subsoil mechanical, hydraulic and thermal features in the Solfatara-Pisciarelli area, our study also highlights the need for detailed subsurface information that could only be obtained via deep scientific drilling^16,17^ and extensive high-resolution 3D geophysical surveys^48,49^. These would allow direct monitoring of thermal anomalies due to deep hot sources and complement geochemical information from fumaroles. Fumaroles are in fact useful “hot control points” that locally spill quasi-isenthalpically the deep hydrothermal system, but whose variations cannot be entirely transposed into the lower-T and water-saturated deforming rock volume.

## Methods

### Thermodynamic analysis of hydrothermal and magmatic fluids

Our main goal is the reconstruction of the temporal evolution of the thermodynamic state of the deep hydrothermal system, particularly its temperature and phase constitution. Its thermal history is determined independently of the geochemical composition of fumarole discharges and based on the C-isotopic exchange between CO_2_ and CH_4_ species. The ensuing thermodynamic analysis shows that deep hydrothermal fluids experience after 1984 a supercritical excursion (with respect to pure water) or evolution to superheated vapour (with respect to a real H_2_O-CO_2_ 80:20 molar mixture), such that the liquid phase disappeared (see also Supplementary information). The locus of P-T-H paths connecting the deep dried hydrothermal system and fumarole vent conditions, through the shallow hydrothermal system, shows that the ascending fluid plume cannot experience steam condensation and follows nearly isenthalpic rising paths. This demonstrates that the increasing CO_2_-fraction observed in fumaroles is determined by the increased percolation and inflow of CO_2_-rich magmatic fluids released by the 8 km deep magma reservoir. Isenthalpic rise of such CO_2_-rich deep magmatic fluids represents the effective heat source responsible of the observed drying of the deep hydrothermal system. The shallow P-T equilibrium conditions of the hydrothermal fluid have been estimated by geothermobarometric functions within the CO_2_-H_2_O-CO-CH_4_-H_2_ system and show that single gas-phase equilibrium conditions take place after 1984 (Supplementary Figure 1). It was already demonstrated^15^ that CH_4_ participates to the full chemical equilibrium at temperatures lower than those recorded by the isotopic exchange between CO_2_ and CH_4_ (Fig. 2c). For CH_4_, rates of chemical and isotopic exchanges are in fact different, such that isotopic equilibration is ~400 times slower than the chemical one^50^ and cannot be recorded by fumarolic gases.

Thermodynamic properties of pure water and ideal H_2_O-CO_2_ mixtures (Fig. 3a,b and Supplementary Figure 2) have been computed by using the web-calculator provided by the National Institute of Standards and Technologies, available at: http://webbook.nist.gov/chemistry/fluid © 2016 by the U.S. Secretary of Commerce on behalf of the United States of America.). Vapor-liquid P-H-T conditions for the real H_2_O-CO_2_ 80:20 (molar) mixture in Fig. 3a,b are from ref.^32^.

#### Computation of isotopic temperatures of the deep hydrothermal system

Because isotopic equilibration is ~400 times slower than the chemical one^50,51^, ^13^C exchange between CO_2_-CH_4_ quenches the thermal condition of CH_4_ formation, hence the thermal condition of the generation of deep hydrothermal system upon interaction with magmatic gases^22^. Based on available measurements of ^13^C exchange between CO_2_ and CH_4_ (Table 1), isotopic temperatures have been computed by using the following thermometer^52^ between 473.15 K and 873.15 K (or 400 °C and 600 °C):

1 $$\begin{array}{rcl}{{\rm{\delta }}}^{13}{{\rm{C}}}_{{\rm{CO2}}} \mbox{-} {{\rm{\delta }}}^{13}{{\rm{C}}}_{{\rm{CH4}}}={10}^{3}{\mathrm{In}{\rm{\alpha }}}_{(\mathrm{CO2} \mbox{-} \mathrm{CH4})} & = & 26.70-49.137\cdot ({10}^{3}/{\rm{T}})+40.828\cdot ({10}^{6}/{{\rm{T}}}^{2})\\  &  & -\,7.512\cdot ({10}^{9}/{{\rm{T}}}^{3})\end{array}$$

Standard deviation is 0.14‰ on 10^3^lnα, where α is defined as:

2 $${{\rm{\alpha }}}_{({\rm{CO2}} \mbox{-} {\rm{CH4}})}=({}^{13}{\rm{C}}{{\rm{O}}}_{2}/{}^{12}{\rm{C}}{{\rm{O}}}_{2})/({}^{13}{\rm{C}}{{\rm{H}}}_{4}/{}^{12}{\rm{C}}{{\rm{H}}}_{4})$$

#### Computation of equilibrium temperatures and pressures of the upper hydrothermal system

In order to compute the temperature of the equilibration zone of gas species within the hydrothermal system (upper hydrothermal reservoir) (Supplementary Figure 1) we adopted the water-gas shift equilibrium^15,53,54^:

3 $${{\rm{H}}}_{2}{\rm{O}}+{\rm{CO}}\iff {{\rm{CO}}}_{2}+{{\rm{H}}}_{2}$$

whose equilibrium constant (1 bar, T of interest standard state) provides a pressure- and redox-independent estimate of equilibrium temperature:

4 $$\mathrm{log}\,\frac{[CO][{H}_{2}O]}{[{H}_{2}][C{O}_{2}]}=-\,2248/T+2.485$$

Terms in square brackets denote thermodynamic activities, which can be replaced by concentrations. Note that equilibrium temperatures, recorded by gas species of the single vapor phase, hold valid also for the liquid phase eventually coexisting with vapor^15^.

From the following reaction^15,54^

5 $${{\rm{CH}}}_{4}+2{{\rm{H}}}_{2}{\rm{O}}\iff {{\rm{CO}}}_{2}+4{{\rm{H}}}_{2}$$

The hydrothermal pressure, P (Supplementary Figure 1), is obtained by reworking on the expression for the equilibrium constant of reaction 5:

6 $$\mathrm{log}\,{P}_{TOT}=\frac{1}{2}\,\mathrm{log}\,\frac{[C{H}_{4}]}{[C{O}_{2}]}\cdot \frac{{[{H}_{2}O]}^{2}}{{[{H}_{2}]}^{4}}+5.38-4661.5/T(K)$$

Partial pressures (i.e., PH_2_O and PCO_2_) can be then computed on the basis of Raoult’s law.

Plotting T vs PH_2_O (Supplementary Figure 1) allows a straight evaluation of the rare attainment of liquid-vapour coexistence, with 1983 Forum Vulcani points falling close to the point of maximum enthalpy of either the pure saturated steam (236 °C, 3.1 MPa) or the saturated 80:20 H_2_O-CO_2_ vapor (~250 °C and ~5 MPa).

#### Isenthalpic paths of deep gas ascent

Isenthalpic deep gas ascent, or irreversible adiabatic decompression (IAD), obeys the following thermodynamic relation^55^:

7 $${(\frac{\partial T}{\partial P})}_{H}\equiv {\mu }_{JT}=\frac{V\cdot (T\alpha -1)}{{C}_{p}}$$

in which *μ*_*JT*_ is the Joule-Thomson coefficient), *V* the partial molar volume, α the therrmal expansivity, and *C*_*p*_ the constant pressure heat capacity. Supplementary Figure 2 shows IAD paths for pure H_2_O, pure CO_2_ and a H_2_O/CO_2_ = 1 mixture rising from the deep magmatic source into the deep hydrothermal system (~25 MPa). These paths are compared to that followed by a steam-rich (H_2_O/CO_2_ = 2.5) magmatic gas released from the shallow decompressing magma hypothesized in ref.^24^ (1000 °C and 120 MPa at the end of the 1984–2004 subsidence phase). At the entrance of the hydrothermal system, all paths end at a temperature compatible with that of magmatic gases (~900 °C; ref.^22^) inferred from independent energetic considerations. In particular, because of the Joule-Thomson effect, the pure CO_2_ gas records a temperature increase due to gas heating on ascent (1055 °C at 25 MPa), whereas the H_2_O-CO_2_ mixture keeps a high temperature (961 °C at the hydrothermal interface, or 25 MPa), that is 100 °C higher than that of pure H_2_O (863 °C) at the hydrothermal interface. The hypothetical steam-rich gas released at 120 MPa, under isenthalpic conditions, would enter the hydrothermal system at 935 °C. Therefore, the H_2_O-CO_2_ 1:1 mixture percolating since year 2000, brings 43.5 J/g ( = *ρC*_*p*_Δ*T*, with Δ*T* temperature difference at 25 MPa between the two gas mixtures) more than the shallow steam-rich magmatic gas released by a shallow magma at 120 MPa. Such a difference makes the deep degassing of a CO_2_-rich supercritical gas energetically more advantageous than the release of steam-rich gas from a shallower magma, which in any case cannot support the degassing process for long-time and soon reaches exhaustion. It is clear that only the persistent continuous influx of the CO_2_-rich deep gas can sustain the observed heating and drying of the hydrothermal bottom, and that in the case of pure CO_2_ infiltration, heating effects would be magnified by the CO_2_ Joule-Thomson effect.

### Modeling of conductive/convective heat transfer after sill emplacement

In order to evaluate the thermal effect of a thin-sill intrusion on the thermal profile of the magma surrounding rocks and of the hydrothermal system, we perfomed simulations of the conductive/convective heat transfer following emplacement by using the Heat3D code^56^. This solves heat flow by finite difference solution of energy and momentum conservation equations (i.e., Navier-Stokes). These equations describe heat transfer by conduction and convection with nonlinearities arising from variation of thermal conductivity in a non-isotropic, heterogeneous, material with heat sources/sinks (e.g., latency). The Boussinesq approximation is employed in the Navier-Stokes equations and momentum conservation by Darcy’s equation is employed because of its empirical success. HEAT3D employs an explicit finite differencing scheme, such that the original differential equation solved is exactly reproduced by the finite difference equation when time and spatial steps are infinitesimal. Truncation errors that might evolve when using very short time steps are minimized by utilizing double precision. Continuous thermal gradients are assigned along the boundaries where symmetry planes are not specified, and initial conditions use a designated regional thermal gradient. The heat advection caused by rock displacement necessary to make space for the magma was considered in our simulations. Used rock/magma properties are given in Supplementary Table 1.

We modelled a 800 × 200 element planar regular mesh (grid size = 25 m), corresponding to 20 km in length and 5 km in depth. For computational limits, it was not possible to decrease the grid size to values comparable with the expected thickness of the intruding sill,that is in the order of one meter (Fig. 2b). Convection was assumed to occur in a hydrothermal system extending over a distance of 4 km from the center of the calculation domain, thus 8 km in total. Two possible depths of the hydrothermal system were considered: 2.5 km (ref.^22^) and 4 km (i.e., along the base of the B’C’D’E’F’G’ quadrangle in Fig. 3a). From previous drillings made by AGIP^34^ we know anyway that the aquifer bottom, i.e. the depth at which the supercritical temperature for water, lies in the whole area in the depth range 2.5-3.0 km. The higher depth of 4 km then maximize the speed of propagation of the heat coming from the sill, because of the convective effects in the aquifer, which is here in contact with the sill.

As thermal boundary condition, we set a thermal gradient of 0.15 °C/m. After 4400 years (i.e., the time elapsed since the last eruption also originating the Solfatara-Pisciarelli system^44^), this condition returns a temperaure-depth profile compatible with geochemistry^22^, as well as borehole thermal gradients^34^.

Then, in our model, a sill, having a thickness corresponding to the grid size (i.e., 25 m), suddenly emplaces at 4 km depth. Supplementary Figure 2 shows that, over a time span of 100 years after emplacement, it produces negligible thermal perturbations in the surrounding hydrothermal system. Even extending the base of the hydrothermal system down to 4 km, i.e. right on top of the sill, does not change appreciably the initial temperature profile. In fact, only the very bottom layer records a sudden temperature increase upon sill-emplacement, from 520 °C to ~570 °C after 20 years, but then remains stable.

Finally, it is worth noting that in both simulations, magma solidus conditions are achieved very quickly (~10–20 year). All above results, all the more so, apply also to a sill thinner than the one modelled here.

### Deformation modelling

The expansion of the water + gas filled rock volume (*V*_*r*_) in response to changes of temperatures and gas content (*n*) within the reservoir is given by:

8 $${\rm{\Delta }}V={V}_{r}-{V}_{0,r}=\int {(\frac{\partial {V}_{r}}{\partial T})}_{P,n}dT+\int {(\frac{\partial {V}_{r}}{\partial n})}_{P,T}dn$$

with *V*_0,r_ the initial volume prior to expansion. Considering that the second term in equation (8) is relevant only within the limited portion of the fumarole area, and that injected gas (i.e., CO_2_) can be entirely considered dissolved in acquifers without appreciable pore volume gain (i.e., along black P-T paths in Fig. 3b), we can focus our attention on the first term in integral. This would produce a volumetric deformation (ε = Δ*V*_*r*_*/V*_*0*,*ρ*_) proportional to *α*_*rock*_ = *ϕα*_*w*_ + (1 − *ϕ*)*α*_*s*_ with *α*_*w*_ the coefficient of (volumetric) thermal expansion of water, α_*s*_ the coefficient of (volumetric) thermal expansion of the solid matrix and *ϕ* the porosity. By using α_*w*_ = 5·10^−4^ °C^−1^, α_*s*_ = 0.5·10^−4^ °C^−1^ and *ϕ* = 0.2 (refs^29^ and ^35^), and for Δ*T* between 1 °C and 5 °C, ε ranges from 0.00014 to 0.0007. In order to translate such volumetric deformation of the hosting rocks (i.e. at the source) into surface deformation, we use here the established relationships between volume expansion at the source volume change (Δ*V*) and surface uplift volume (U*V*), which depend on the source model assumed^56^. For a sill-like source, the volume expansion at the source is equal to the uplift volume at surface, whereas for a point-source model^57^ Δ*V* = U*V/2*(1 *−* *ν*), where *ν* is the Poisson coefficient of the host rock. Since we are modelling a source involving a rather large volume of the heated porous medium, we can approximate the source model to a point-source model, because it is well known^58^ that the point source approximation works well also for non negligible *r/d* ratios (where *r* is the radius of a sphere undergoing volume or pressure changes, and *d* is the depth of its centre). Assuming a spherical source with center at 3.0 km of depth (*d*), a radius of 2 km (i.e. *V* = 33.5·10^9^ m^3^), we can compute the volume increase, using both the values of ε computed for the limiting temperature changes of 1 °C and 5 °C, as Δ*V* = ε·*V*. We obtain a value Δ*V* = 47.3·10^5^ m^3^ for Δ*T* = 1 °C, and Δ*V* = 236.6·10^5^ m^3^ for Δ*T* = 5 °C. The maximum vertical surface displacement (i.e., just above the source) resulting from the volume change Δ*V* is *h*_max_ = Δ*V*(1 − *ν*)/(π*d*^2^) (ref.^59^). Using a value of *ν* = 0.25, the resulting maximum displacement ranges between 0.13 m (for Δ*T* = 1 °C) and 0.65 m (for Δ*T* = 5 °C), which compares well with an observed value of 0.40 m. In principle, the source could be centred at lower depth: as an example, a source centred at 2 km, with a radius of 1.5 km (i.e., *V* = 14.1·10^9^ m^3^), would produce almost exactly the same values. The initial rock volume so far interested by deformation would then be on the order of ~15 to ~35 km^3^.

### Data availability

Solfatara fumaroles data are fully available from refs^15,22–25,53^. Supplementary data file reports the full listing of composition of the Pisciarelli fumarole, sampled since 1999. For this fumarole, data until September 2009 can also be found in ref.^53^. In all cases, concentrations are expressed in μm/mol and analytical methods are reported in ref.^22^. Seismological data (magnitudo and hypocenters depth can be found on the *Plinio* seismic data repository (http://www.ov.ingv.it/ov/en/banche-dati.html) or from INGV-OV bulletins (http://www.ov.ingv.it/ov/en/campi-flegrei/275.html). All data, computations and time series are available on request to the corresponding author.

## Electronic supplementary material

SUPPLEMENTARY INFORMATION
