# Supplementary material for: Caldera unrest driven by CO2-induced drying of the deep hydrothermal system
Source: Sci Rep. 2018 May 29;8:8309. doi: 10.1038/s41598-018-26610-2 (PMC5974283; doi:10.1038/s41598-018-26610-2)
Supplement: Supplementary file 1 — SUPPLEMENTARY INFORMATION [file 41598_2018_26610_MOESM1_ESM.pdf]

SUPPLEMENTARY INFORMATION TO THE PAPER:

"Caldera unrest driven by CO_2-induced drying of the deep hydrothermal system"

R. Moretti, C. Troise, F. Sarno and G. De Natale

Supplementary Table 1

TABLE S1. Rock and magma properties used in simulations of conductive/convective heat trasfer. HEAT3D[54] default values of thermal conductivity (K) and heat capacity (Cp) are used. Rock porosity from ref. 35; rock density from ref. 36; magma density and initial magma temperature (T_0) from ref. 43.

**Rock type**	**K (W/m K)**	**Cp (J/kgK)**	**Density (kg/m^3)**	**porosity**
**Tuff**	1	980	2000	0.2
**Sill intrusion**	**K (W/m K)**	**Cp (J/ kg K)**	**Density (kg/m^3)**	**T_0 (°C)**
**trachyte**	1.3	1150	2750	900

Supplementary figure 1 and caption

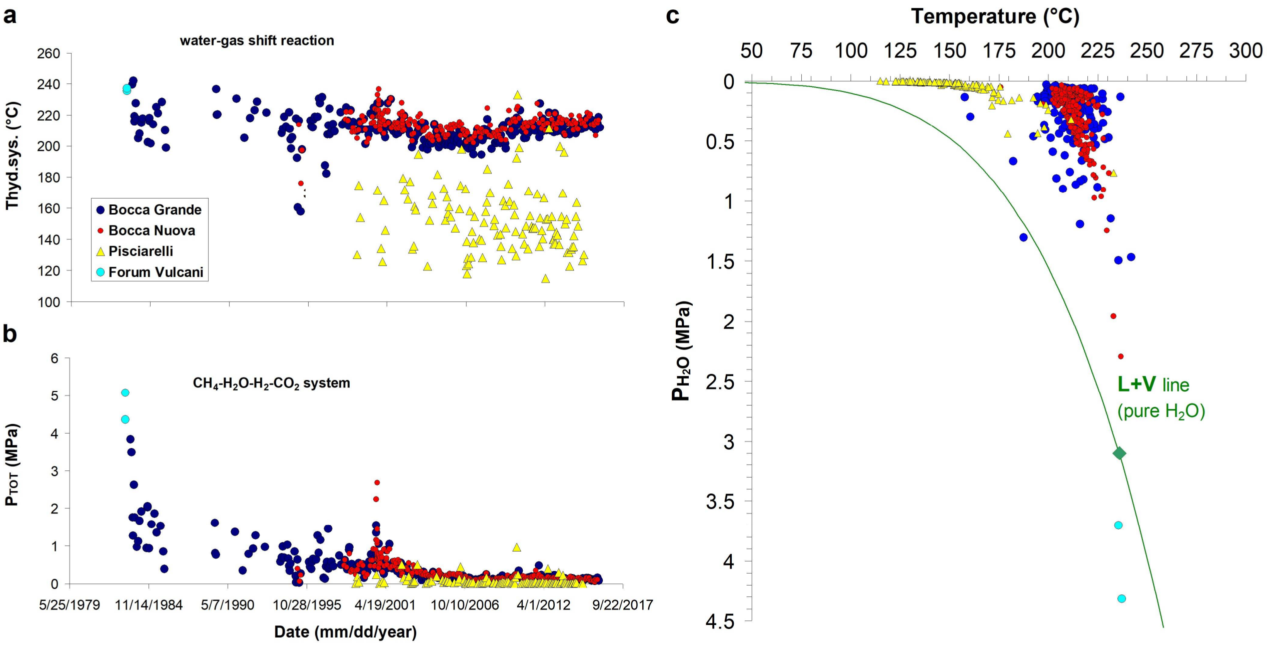

Supplementary figure 1 | P-T conditions of the upper hydrothermal reservoir. a. Chronogram of computed shallow hydrothermal equilibrium temperatures at Solfatara and Pisciarelli vents by using the water-gas shift reaction (equation 2; see Methods). Note that the Pisciarelli system does not show any appreciable heating, whereas Solfatara hydrothermal temperatures draw a minimum around year 2007. **b.** Chronogram of pressures computed from thermodynamic equilibrium involving CO_2-H_2O-H_2-CH_4 species in the single gas phase (equation 4; see Methods). **c.** P-T diagram comparing results on the shallow hydrothermal system to the liquid-vapour equilibrium of pure water (see Methods).

Supplementary figure 2 and caption

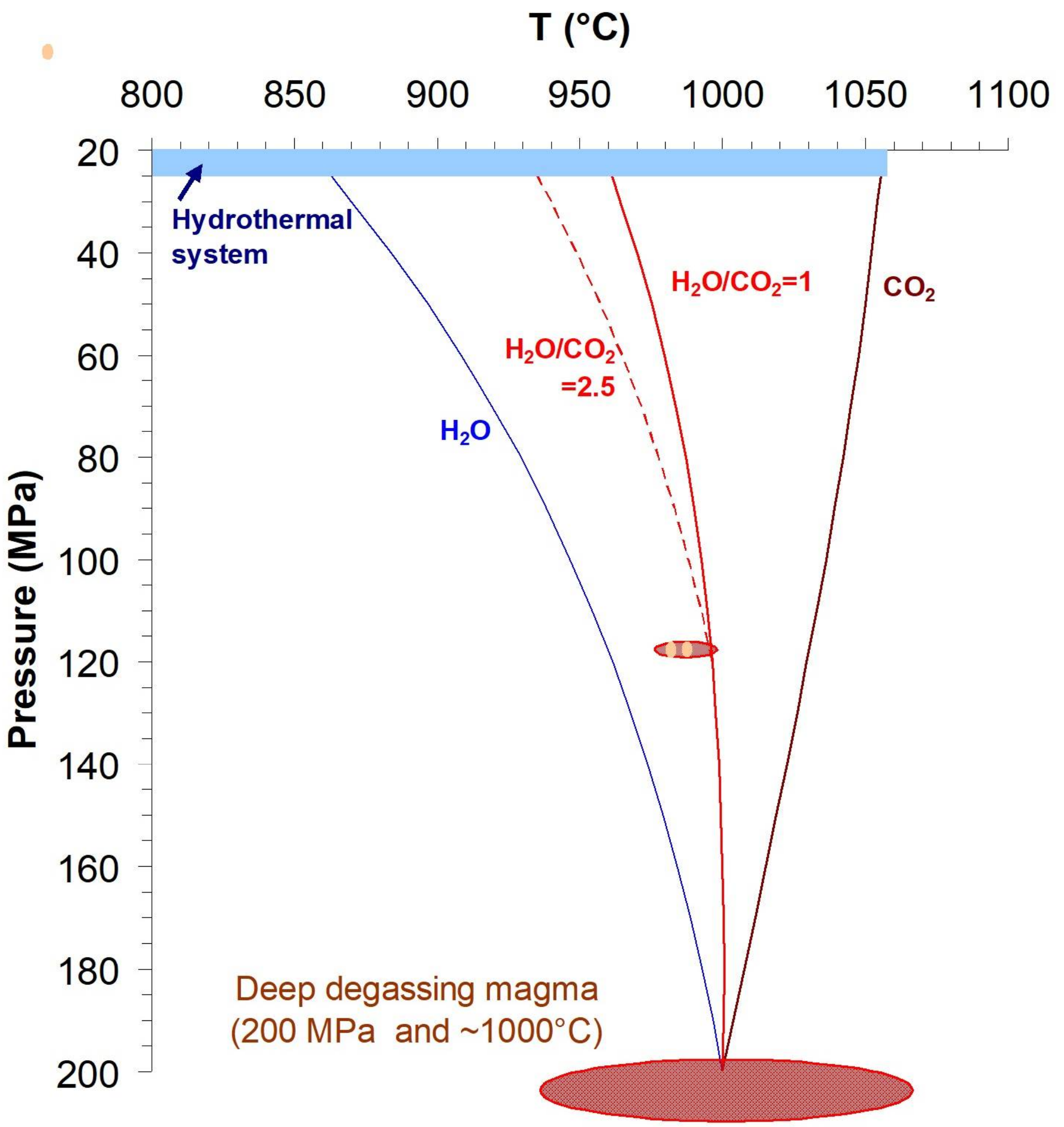

Supplementary figure 2 | P-T properties of the deep fluids. P-T diagram reporting IAD paths (eq. 5) of deep magmatic gas, computed from 200 MPa and 1000°C to 25 MPa (for pure-H_2O, pure CO_2, and 1:1 H_2O-CO_2 mixture) and from 120 MPa and 1000°C to 25 MPa (2.5:1 H_2O-CO_2 mixture, as in ref. 24). The blue stripe between 20 and 25 MPa indicate conditions attained by the gas phase right prior to interact with the hydrothermal component to form the gas plume subsequently discharged at fumaroles[22,23]. The Joule-Thomson coefficients (μ_{JT}) for pure components were linearly combined (i.e., ideal mixing in the P-T range of interest) in case of 1:1 and 2.5:1 H_2O-CO_2 mixtures (see Methods).

Supplementary figure 3 and caption

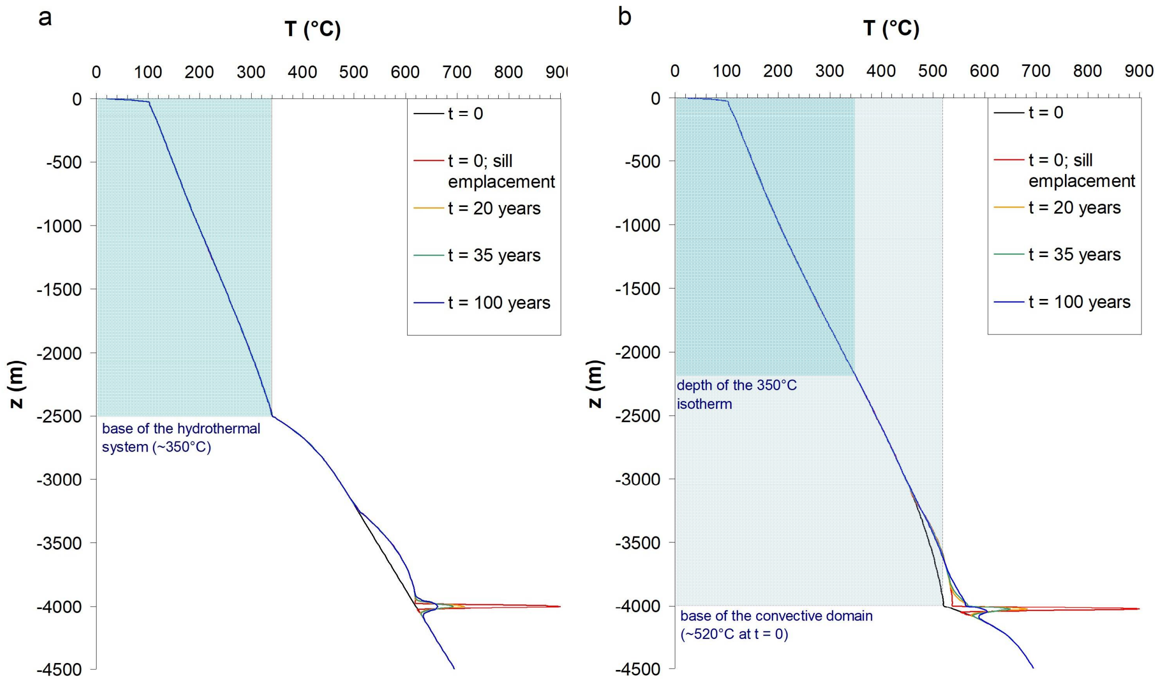

Supplementary figure 3 | Results of conductive/convective modeling[56] of thin-sill emplacement at 4 km depth. a. T depth profile for hydrothermal system having its base at 2.5 km at a temperature of ~ 350°C (ref. 22). **b.** T-depth profile of a convective domain extending down to 4 km, that is, right on top of the emplacing 25 m-thick sill. Note that in this second case the 350°C isotherm is shifted ~ 250 m upward.

Arguments against a persistent shallow degassing magma

Several geochemical arguments play against a shallow magma that drives the current unrest at CFc by relaxing its overpressure since 1984 and releasing steam upward[24,25]:

1) CO_2 plus inert-gas (N_2, He, Ar) degassing patterns modelled in refs. 24,25 hold only for a primary trachybasalt or shoshonite compositions, the least represented compositions of CFc volcanism[45]. However, it is unrealistic that a trachybasaltic may evolve uniquely as a gas+melt system up to shallow depths.

2) A persistent shallow magmatic source would be conceptually according to some geophysical inversions of geodetic and seismic data[S1-S3], but overpressure relaxation from modelled degassing patterns is such that pressure was 200 MPa in 1984 and is 120 MPa in 2014 (ref. 24), resulting in an initial overpressure of 80 MPa. This values largely ovecomes the tensile strength of the overlying crustal rocks[13,26] and can be compatible only with a lithostatic load of about 7-8 km. Thus, if a shallow magma were triggering the current uplift, the 1982-84 unrest was hydrothermal[22], in agreement with temporal trends of geochemical indicators, which point to different triggering mechanisms for these to episodes (Fig. 2a).

3) It is well established that steam is extracted from magma via “second boiling” and crystallization [23,S4,S5], which causes the downward migration of a growing solid carapace surrounding the liquid residuum and then leads to a scenario completely opposite to that of a shallow decompressing magma.

4) Independently of the source of juvenile steam (magma second boiling vs continuous decompression), the amount of magma needed to sustain the steam flux of ~ 3500 tons/day (ref. 24) is not compatible with available geophysical estimates. If we consider that the amount of magma possibly emplaced in 1982-84 occupies at most the same volume as that of the inflation corresponding to maximum displacement (0.09 km^3, see Methods), and using a density of 2500 kg/m^3, steam flux could have been sustained for 17.5 years at most (i.e., until 2002) before

exhaustion, even under most favourable conditions, i.e., adopting the highest H_2O content CFc magmas (4 wt%; ref. 44) and considering that ~40% of discharged steam comes from magma degassing[22,23].

These simple arguments further dismount shallow magma hypotheses for the present unrest and play in favour of a deep and persistent gas contribution (H_2O/CO_2 ratio between 1 and 1.5) from the 8 km-deep magma regional reservoir[16,23,44,45,].

On the nature of the deep hydrothermal system

Lacking a shallow degassing magma, the observed thermal evolution can be ascribed to either 1) drying of the hydrothermal low-saline liquid, now replaced by a high-T supercritical gas or vapor, or 2) increasing salinity of the hydrothermal boiling liquid, now replaced by a NaCl brine like at the nearby Vesuvius volcano[S6]. In the first case the increasing CO_2/H_2O fumarole ratio accompanies decreasing involvement of the hydrothermal end-member and the relative increase of the CO_2-rich deep gas contribution[16,23], whereas in the second case it may be related to CO_2-solubility drop in a brine (salting-out effect[S1,S2]). In this latter scenario, temperatures in Fig. 2c would suggest that the brine boiling point increases with increasing salt concentration due to the progressive neutralization of acid magma gases. Transition to a brine may thus have occurred rapidly at the end of previous hot cycles (e.g., between 30 and 40 years at Vesuvius[S1]). We suggest however that the first scenario is the only one plausible, due to the very high CO_2-concentrations (Fig. 2a) and magmatic fluid fractions (> 0.4; refs. 22,23) and because it easily accounts for high rates of gas discharges at surface and the widening of the CO_2 degassing area[24]. On the other hand, the second scenario should lead to progressive cooling of the shallow system and much lower magmatic gas fractions, neither of them observed.

Supplementary references

S1. Amoruso, A., Crescentini L. & Sabbetta, I. Paired deformation sources of the Campi Flegrei caldera (Italy) required by recent (1980–2010) deformation history. *Journal of Geophysical Research: Solid Earth* **119**, 858-879 (2014).

S2. Amoruso, A. *et al.* Clues to the cause of the 2011–2013 Campi Flegrei caldera unrest, Italy, from continuous GPS data. *Geophysical Research Letters* **41**, 3081-3088 (2014).

S3. De Siena, L. *et al.* 4D imaging of the seism-geochemical dynamics leading to recent Campi Flegrei unrest. Geophysical Research Abstract 19, EGU2017-1877. EGU General Assembly 2017, Vienna (2017).

S4. Bodnar, R.J. *et al.* Quantitative model for magma degassing and ground deformation (bradyseism) at Campi Flegrei, Italy: Implications for future eruptions. *Geology* **35**, 791-794 (2007).

S5. Lima, A. *et al.* Thermodynamic model for uplift and deflation episodes (bradyseism) associated with magmatic–hydrothermal activity at the Campi Flegrei (Italy). *Earth-Science Reviews* **97**, 44-58 (2009).

S6. Chiodini, G., Marini, L. & Russo, M. Geochemical evidence for the existence of high-temperature hydrothermal brines at Vesuvio volcano, Italy. *Geochimica et Cosmochimica Acta* **65**, 2129-2147 (2001).

S7. Truesdell, A.H. Origins of acid fluids in geothermal reservoirs. *Geotherm. Res. Counc. Trans.* **15**, 289 –296 (1991).
